# Drugs Used in “Chemsex”/Sexualized Drug Behaviour—Overview of the Related Clinical Psychopharmacological Issues

**DOI:** 10.3390/brainsci15050424

**Published:** 2025-04-22

**Authors:** Fabrizio Schifano, Stefania Bonaccorso, Davide Arillotta, Amira Guirguis, John Martin Corkery, Giuseppe Floresta, Gabriele Duccio Papanti Pelletier, Norbert Scherbaum, Nicolò Schifano

**Affiliations:** 1 Psychopharmacology, Drug Misuse and Novel Psychoactive Substances Research Unit, School of Life and Medical Sciences, University of Hertfordshire, Hatfield AL10 9AB, UK; f.schifano@herts.ac.uk (F.S.); j.corkery@herts.ac.uk (J.M.C.); gducciopapantip@gmail.com (G.D.P.P.); 2 North Islington Core Team, Academic Secretary RCPsych, London N7 8US, UK; stefania.bonaccorso2@nhs.net; 3 Department of Neurosciences, Psychology, Drug Research and Child Health, Section of Pharmacology and Toxicology, University of Florence, Viale G. Pieraccini, 6, 50139 Florence, Italy; 4 Pharmacy, Medical School, The Grove, Swansea University, Swansea SA2 8PP, UK; amira.guirguis@swansea.ac.uk; 5 Department of Drug and Health Sciences, University of Catania, 95124 Catania, Italy; giuseppe.floresta@unict.it; 6 Tolmezzo Community Mental Health Centre, ASUFC Mental Health and Addiction Department, Via Bonanni 2, 33028 Tolmezzo, Italy; 7 Department of Psychiatry and Psychotherapy, LVR-University Hospital Essen, Medical Faculty, University of Duisburg-Essen, 45147 Essen, Germany; norbert.scherbaum@lvr.de; 8 Department of Urology, ASST Sette Laghi-Ospedale di Circolo di Varese, 21100 Varese, Italy; schifanonicolo90@gmail.com

**Keywords:** chemsex, sexualized drug behaviour, drug use, substance misuse, drug addiction

## Abstract

**Background**: “Chemsex” involves the intake of a range of drugs (e.g., synthetic cathinones, gamma-hydroxybutyric acid/gamma-butyrolactone (GHB/GBL), ketamine, methamphetamine, “poppers”, type V phosphodiesterase (PDE) inhibitors, MDMA/ecstasy, cocaine, cannabis, and occasionally a few other molecules as well, to enhance and prolong sexual experiences. This paper aims to provide an overview of the clinical pharmacology of the vast range of drugs that are being used for chemsex with a focus on both the medical and psychopathological disturbances that they can produce. **Methods**: A narrative literature review was conducted using Pubmed, Scopus, and Web of Science databases. A total of 273 papers published up to January 2025 were screened; articles were selected based on relevance to chemsex/sexualized used behaviour and related substances. Both human and preclinical studies were considered. **Results**: The use of stimulants is likely related to the need to increase as much as possible both sexual arousal and performance but also to increase social interactions. Furthermore, the empathogenic/entactogenic activities of some MDMA-like “love drugs” facilitate the occurrence of “feeling closer/more intimate” emotional sensations, and GHB/GBL may provide the user with a subjective sensation of disinhibition, hence facilitating condomless meetings with a higher number of random partners. Conversely, ketamine may be used to both enjoy its psychotropic dissociative characteristics and facilitate the potentially painful receptive anal intercourse and/or fisting experiences. Most typically, these drugs are consumed in combination, with polydrug exposure possibly facilitating the occurrence of serotonergic syndrome, seizures, drug–drug pharmacokinetics’ interaction, and sympathomimetic overstimulation. Following these polydrug exposures, a range of psychopathological conditions have at times been reported. These issues may lead to misuse of opiates/opioids, gabapentinoids, and/or antipsychotics. **Conclusions**: Further actions should aim at reducing the stigma that prevents individuals from accessing necessary healthcare and support services. A multidisciplinary approach that combines medical, psychological, and social support remains key to managing the complex challenges posed by chemsex-related drug use.

## 1. Introduction

“Chemsex” has been defined as a voluntary intake of certain psychoactive and non-psychoactive drugs in the context of sex parties and sexual intercourse with the intention of enhancing, prolonging, and sustaining sexual experiences, hence facilitating the sexual encounter [1]. These sessions are mostly among men who have sex with men [2,3,4,5,6,7,8,9,10,11,12,13,14]. Conversely, one could argue that the use of drugs whilst being involved in intimate, sexual behavior may relate to a vast range of populations. From this point of view, similar concepts have been proposed, including perisexual drug use [15], sexualized drug behavior [16], “Sex on Chems” (Wilson and Williamson, 2024) [17], “Party-n-Play” [18], wired sex [19], and “Pampalibog” (in the Filipino language; [20]). Chemsex practices are becoming increasingly popular. Data from 55, 446 MSM subjects living in 44 urban centers were made available by Schmidt et al. [21]. In the European region, the past 4 weeks’ chemsex involvement was highest in Brighton (16.3%), Manchester (15.5%), London (13.2%), Amsterdam (11.2%), and Barcelona (7.9%). In 2018, 785 MSM were recruited at nine Dutch clinics, and 511 (65%) completed the online questionnaire. Chemsex, which was defined as using cocaine, crystal meth, designer drugs, gamma-hydroxybutyric acid/gamma-butyrolactone (GHB/GBL), ketamine, “speed” and/or 3,4-methylenedioxymethamphetamine (XTC; MDMA), was reported by 41% of interviewees during the previous 6 months [22].

At times, to enhance libido, potency, and sexual pleasure, a range of aphrodisiacs, which are often naturally occurring and traditionally used, are self-administered [23]; moreover, aphrodisiacs’ intake is frequently rooted in cultural beliefs and superstitions. The U.S. Food and Drug Administration (FDA) defines an aphrodisiac drug product as “any product that bears labeling claims that it will arouse or increase sexual desire, or that it will improve sexual performance”. Conversely, the European Medicines Agency (EMA) does not have specific definitions for aphrodisiac products but regulates drugs for sexual dysfunction [24]. As a result, a range of over-the-counter (OTC) herbal products and approved prescription drugs (for example, for erectile dysfunction) are legally available. From an anthropological perspective, chemsex cultures are multifaceted and shaped by a wide range of social and psychological factors [25], for example, geographical location, the use of apps and online platforms, and the availability of specific substances. Indeed, there is a lack of research as well on how sexual experience affects drug reward in animals [26]. Mating is clearly regarded as a basic reward activity. In fact, conventional reinforcers (e.g., food, sex) stimulate dopamine (DA) transmission in the nucleus accumbens shell [27]. Addictive drugs share with conventional reinforcers the property of stimulating DA transmission in the nucleus accumbens shell. This response, however, undergoes one-trial habituation in the case of conventional reinforcers. Resistance to habituation allows drugs to repetitively activate DA transmission in the shell upon repeated self-administration [27]. In sober sex, a clear post-ejaculation refractory time (PERT), e.g., the period after a single ejaculation when further erections and ejaculations are inhibited, has been documented [28]. Conversely, Schreck et al. [29] documented a 40-hour-long chemsex session. Further, a recent UK-based, mixed methods study surveyed some 123 subjects; 86% of respondents engaged in riskier sex during sessions and 35% no longer enjoyed sober sex [17]. Hence, one could argue that chemsex may respond to the perceived need to use a range of drugs to overcome resistance to habituation, synergistically increase sex-derived physiological pleasure, and facilitate voluntary self-exposure to idiosyncratic practices. To experience a more intense rush and longer sex, some drugs are being injected so that much higher bioavailability levels are being attained [30,31].

In considering the above, this paper aims to provide an overview of the clinical pharmacology of the vast range of drugs that are being used for chemsex, with a focus on both the medical and psychopathological disturbances that they can produce.

## 2. Methodology

For this narrative review, a literature search was performed using Pubmed, Scopus, and Web of Science databases from inception until January 2025 through the following search strategy: (“chemsex”, OR “perisexual drug use”, OR “sexualized drug behavior”, OR “sex on chems”, OR ”party-n-play”, OR ”wired sex”) AND (“GHB/GBL”, “synthetic cathinones”, “ketamine”, “amphetamine-type substances”, “MDMA”, “poppers”, “type V phosphodiesterase (PDE) inhibitors”, “cannabis”, “cocaine”, “alcohol”). Particular focus was here as well on the above substances’ associated medical and psychiatric manifestations. Evidence included in the review comprised both human data and preclinical data, if and when this was of interest. Each article’s title and abstract were reviewed for their appropriateness with regard to the relationship between chemsex/sexualized drug behavior and the specific classes of substances highlighted here, including alcohol. In this way, some 273 papers were initially scrutinized for relevance. Although the papers here quoted were published in the time frame 1986–2025, some 6 out of 10 of them were published in the period 2020–2025.

## 3. The Drugs Being Used in Chemsex Scenarios, Rationale for Their Use, and Related Risks

### 3.1. Amphetamine-Type Stimulants (ATS); “Meth” and “Crystal Meth”

Amphetamine-type substances include oral (“meth”), smokable (“crystal meth”) and injectable/rectally administered methamphetamine, together with a vast range of other substances, including PMA (4-methoxyamphetamine, “Dr. Death”), PMMA (4-methoxymethamphetamine), 4-MTA (4-methylthioamphetamine, “flatliners”), DMA (2,5-dimethoxyamphetamine), MPA (methiopropamine), etc. [32]. Meth is the default substance associated with chemsex among MSM subjects in Asia, followed by GHB/GBL and ketamine [33]. Furthermore, Schecke et al. [34] carried out an online survey with some 1050 German-speaking participants who provided information on substance use. They found that 27% used crystal meth in the last 12 months. Some 89% of those subjects used methamphetamine in a sexual setting, and 50% reported injecting this molecule.

Meth acts on DA neuron terminals projecting from the ventral tegmental area to the nucleus accumbens, representing the reward system in the brain [35]. Indeed, there are differences in bioavailability levels between the different methamphetamine formulations; when injecting the molecule, the bioavailability (BA) levels will equal to 100%; with the smokable formulation, the BA levels exceed 90%, and with the oral meth-related BA levels may be of around 60% [36].

Rationale for use: Since ATS stimulant molecules are strong DA agonists, their sex-related ingestion is likely to synergistically activate the DA reward pathways. Meth is being used to maximize both arousal and sexual performance [15]; furthermore, meth ingestion has been associated with feelings of love/sociability [37].

Risks: ATS ingestion may be associated with euphoria, a sense of full energy but also a significant rise in body temperature and death; fatalities are particularly associated with seizures and coma [38]. The main action of stimulants is on the cardiovascular system due to their short- and long-term stimulation of the adrenergic system and consequent effects on blood pressure and myocardial ischemia (for a thorough review, see [39]). Conversely, a chronic ingestion of ATS-related drugs is typically associated with dependence, chronic depression, anxiety, tendency to suicide, and drug-related psychosis [40].

### 3.2. MDMA and the MDMA-like Compounds; the “Love Drugs”

MDMA (3,4-methylenedioxymethamphetamine; ‘ecstasy’; “Molly”, etc.) use is not uncommon in sexualized drug use [41,42].

MDMA is not the only psychedelic phenethylamine (PIAs) molecule; others include products such as the “fly” drugs, the toxic N-methoxybenzyl (NBOMe) compounds, indanes, and benzofurans. Recent and popular appearances in the drug scenario include several 2C-molecules, such as 2,5-dimethoxy-4-bromophenethylamine (2-CB, “Nexus”), 2,5-dimethoxy-4-iodophenethylamine (2C-I), and 2,5-dimethoxy-4-ethylphenethylamine (2C-E). Overall, the range of MDMA-like compounds, including the NBOMes, may be purposefully or unintentionally ingested as MDMA substitutes. Most MDMA-like molecules show agonism at 5-HT2A receptors, whilst some of them also inhibit the dopamine/noradrenaline/serotonin reuptake (for an overview, see [43]).

Rationale for use: With MDMA and MDMA-like drugs, enhanced mood, increased energy, openness, and perceptual alterations are typically reported. These substances are considered both empathogenic and entactogenic, providing the user with a subjective sensation of both disinhibition and “feeling closer/more intimate”; as a result, they are called ‘love drugs’ [44].

Risks: With MDMA and MDMA/like substances, a range of serotonergic and sympathomimetic toxicity effects have been reported [43,45]. Levels of serotonergic toxicity may be severe when MDMA is combined with further serotonergic recreational drugs and SSRIs. At times, after these molecules’ acute ingestion, liver toxicity, panic attacks, mood disorders, acute renal failure, hyperthermia, and even fatalities, as well, can occur [46]. Users have described “mid-week blues” (e.g., fatigue, depressed mood, and decreased appetite) appearing three to five days after the use of ecstasy, and likely, to be associated with depleted serotonin levels [47]. Conversely, severe toxicity and health-related effects have been reported in association with NBOMe compound intake [48].

### 3.3. GHB-like Compounds

Gamma-hydroxybutyrate (GHB; “liquid ecstasy”) and gamma-butyrolactone (GBL) are part of the top drug of the “4-chems”, which include as well, ketamine, crystal meth, and mephedrone/synthetic cathinones [21]. GHB is synthesized exogenously using a relatively simple synthesis with readily available and inexpensive source materials starting from 1,4-butanediol (1,4-BD) or GBL, with both GBL and 1,4-BD being rapidly converted to GHB. Both these GHB precursors are industrial chemicals, and GBL is indeed a high lipophilicity/high potency GHB pro-drug.

The half-life of GHB is <1 h, hence the need for oral redosing [49].

GHB intake is associated with both increased central dopamine levels and activation of GABA-A/B receptors. Euphoria and calmness are initially observed after ingestion [50,51].

Rationale for use: Disinhibitory agents, and GHB/GBL in particular, help the user to achieve a state of relaxation. According to [52], being HIV-positive, having more gay friends, greater social engagement with gay men who use drugs, a greater number of sexual partners, group sex, and condomless anal intercourse with casual partners were all factors independently associated with GHB use in the past 6 months.

Risks: GHB is highly addictive, with its withdrawal syndrome being characterized by insomnia, muscular cramping, tremors, and anxiety. In these cases, inpatient admission is advisable, with high dosages of both GABA-A (e.g., diazepam) and GABA-B (e.g., baclofen) agonists being needed to treat the withdrawal symptoms [32]. A low/moderate oral dose of 10 mg/kg (0.75 g) can produce short-term amnesia, hypotonia, lowering of inhibitions, and libido increase. Higher dosages lead to drowsiness, nausea, vomiting, muscle stiffness, dizziness, confusion, delirium, hallucinations, convulsions, and cardiopulmonary depression (for a thorough review, see Corkery et al. [50]). From 1995 to September 2013, 159 UK-based GHB/GBL-associated fatalities were reported; most deaths (79%) were accidental, and GHB/GBL alone was implicated in 37% of cases [50]. A lethal GHB dose may be in the region of 60 mg/kg [53]. It has been reported that GHB-associated deaths are at least in part due to the increasing use of GHB for chemsex [54].

### 3.4. Synthetic Cathinones

Within the chemsex context, and especially so in the MSM sub-population, intravenous synthetic cathinone misuse has been described in association with methamphetamine and/or GHB/GBL. This scenario may include unprotected sex practice with an average of five sexual partners per session, sharing syringes, and a highly elevated risk of the spread of blood-borne/sexually transmitted diseases (for a review, see Giorgetti et al. [55]).

Overall, an excess of some 180 synthetic cathinones has been [56]. Synthetic cathinones are typically sold as pills, capsules, and powders. They are usually snorted/sniffed (insufflated), taken orally by “bombing” (swallowing the powder wrapped in a cigarette paper), anally administered, mixed in a drink, or through intravenous injection [57]. Mephedrone possesses a re-dosing risk due to a half-life as short as 1 h; in contrast, 3,4-methylenedioxypyrovalerone (MDPV) is thought to have a half-life of 3–5 h [58].

Cathinones have been classified on the basis of their pharmacological action (e.g., dopamine-DAT; serotonin-SERT; norepinephrine transporter-NET inhibition ratio) and comparability with traditional drugs of abuse, in particular:

1. Cocaine/MDMA-mixed cathinones: These act as substrates for dopamine (DAT), serotonin (SERT), and noradrenaline (NET) transporters. Examples include mephedrone, 4-MEC, methylone, ethylone, butylone, and naphyrone. When taken orally, they produce entactogenic, MDMA-like effects; when administered intranasally, they produce cocaine-like effects [59].
2. Methamphetamine-like cathinones: These compounds exhibit high inhibitory potency at DAT and lower inhibitory potency at SERT [60]. Examples include methcathinone, flephedrone, ethcathinone, and 3-fluoromethcathinone (3-FMC).
3. MDMA-like cathinones: These exhibit greater inhibitory potency at SERT than DAT; related molecules include 4-trifluoromethylmethcathinone and methedrone [60].
4. Pyrovalerone-cathinones: These have inhibitory potency at DAT and NET equal to or greater than that of cocaine or methamphetamine [60]: examples include pyrovalerone, MDPV, 3,4-methylenedioxy-α-pyrrolidinohexanophenone (MDPHP), and α-pyrrolidinovalerophenone (α-PVP) [61].

Overall, those synthetic cathinones being specifically considered for chemsex include: mephedrone; methylenedioxypyrovalerone, 4-methylethcathinone, metaphedrone, α–pyrrolidinopentiophenone; N-ethyl-pentedrone (NEPD); α-pyrrolidinohexiophenone; α-PVP; eutylone [62]; 4-methylpentedrone (4-MPD; [63], and methylone. Conversely, most common cathinones being slammed include 3-methylmethcathinone (3-MMC), methamphetamine, ketamine, 4-methylethcathinone (4-MEC), and mephedrone (4-MMC) [64].

Rationale for use: Synthetic cathinones are used in sexualized drug behavior for a variety of reasons, including euphoria, stimulation, increased energy, empathy, openness, mood enhancement, and increased libido. With some synthetic cathinones, as discussed above, marked empathogenic/entactogenic properties are being experienced. These reported “feeling closer to the others” sensations may be particularly appreciated by chemsex enthusiasts since these practices may well involve intimate encounters with previously unknown subjects [55].

Risks: Most frequent unwanted effects after the ingestion of synthetic cathinones include excessive sweating, headaches, palpitations, nausea, cold blue fingers/toes, bruxism, nose bleeds, blurred vision, palpitations, hyperthermia, renal failure, rhabdomyolysis, elevated creatine kinase and hypokalaemia, acidosis, and serotonin syndrome and (for a thorough review (see [65]. Psychiatric unwanted side effects may, at times, include aggression, combative behavior, confusion, paranoid ideation, delusions, hallucinations, paraesthesias, self-harm, and harm to others [32,66].

A range of synthetic cathinones (including mephedrone, methylone, butylone, ethylone, α-PVP, MDPV, MDPHP, and methedrone) has been associated with fatalities, with a large number of them having been associated with hangings and mechanical suicides [67,68].

Synthetic cathinones are strongly addictive, and tolerance levels may develop rapidly [65]. Depression, anxiety, tiredness, insomnia, nasal congestion, and impaired concentration have been described as symptoms indicating mephedrone withdrawal [69]. Paranoid ideation and mood disturbances have been observed in chronic users of synthetic cathinones [57]. Finally, within the chemsex scenario, cathinones may frequently be observed in association with both hepatitis C and HIV [70].

### 3.5. “Poppers”

Some 836 lesbian, gay, bisexual, transgender, queer, intersex, and asexual/LBGTQIA+ subjects were recently surveyed in Belgium; some 258 (30.9%) self-reported having been involved in chemsex practices over the previous 6 months. Most popular drugs included poppers (73%), GHB/GBL (69%), and cathinones (68%) [71]. Some 2919 MSM subjects were surveyed as well in Barcelona, Spain: most frequently used drugs for sexualized drug use were poppers (53.6%; [72]. Popper-related molecules include amyl, butyl, cychloexyl, isobuthyl nitrite (e.g., amy, high-tech, kix, liquid gold, locker, etc.). The alkyl nitrite poppers act as potent vasodilators; some poppers’ effects include a “rush”, “high”, “euphoria”, or feeling of excitement [73]. Furthermore, relaxation of smooth muscles is observed.

Rationale for use: poppers’ ubiquity, and hence ease of access, in chemsex scenarios; because of anal smooth muscles’ sphincter relaxation, facilitation of receptive anal sex intercourse is being reported [73].

Risks: Adverse effects include tachycardia, migraine headaches, fainting, dizziness, and ventricular fibrillation. Both near misses [74] and fatalities have been, at times, associated with either inhalation or ingestion of nitrites [73].

### 3.6. Ketamine and Related Compounds

Dissociative drugs, including ketamine hydrochloride (“special K”) are both popular and a cause of clinical concern [75]. Ketamine-related hallucinogenic/dissociative effects are related to central 5-HT2A agonism, NMDA receptor antagonism, and high affinity for mu/delta/sigma opioid receptors [32]. When misused, ketamine can be injected, snorted, smoked, or administered rectally in a dosage range of 25–300 mg. Its psychotropic effects include referential thinking, dissociation, depersonalization, psychotic experiences, and out-of-body/near-death experiences (e.g., the “K-hole”, [76]. Further ketamine-like drugs include methoxetamine, eticyclidine/PCE, 3-methoxyphencyclidine/3-MeO-PCP, ethylketamine, 3-hydroxyphencyclidine/3-HO-PCP, diphenidine, methoxphenidine/MXP, etc. [32].

Rationale for use: Apart from its recreational, party-related, value, with ketamine and remaining derivatives possessing analgesic/anaesthetic properties, the chemsex-related experiences of receptive anal intercourse and/or fisting may be facilitated [77].

Risks: Long-term recreational ketamine use may present with both urological “k bladder” (e.g., dysuria, hematuria, decreased bladder capacitance) and intestinal (“k cramps”) problems [78]. Although one could argue that people using ketamine in the context of sexual activity are likely to avoid those doses that could make them unable to engage in sex-related activities, high dosage self-administration may be associated with both cardiovascular and respiratory toxicity. Numbness, muscle weakness, and impaired perception can result in falls, trauma, or burns. Ketamine-related risks, not necessarily related to chemsex practices, have also included drowning, death from hypothermia due to lying outside in winter, traffic accidents, and becoming a crime victim (e.g., “trapped in the K-hole”; [75].

From the psychopathological point of view, ingestion of ketamine and its derivatives may be associated with both psychotic manifestations and dissociative states, e.g., out-of-body experiences, and aggression. In the long term, tolerance, dependence, withdrawal signs and flashbacks are described, with schizotypal symptoms and perceptual distortions possibly persisting after cessation (for an overview, see Corkery et al. [75]).

### 3.7. Type V Phosphodiesterase (PDE) Inhibitors

Type V phosphodiesterase (PDE5Is) inhibitors include sildenafil, tadalafil, and vardenafil. Indeed, erection results from the production of cyclic guanosine monophosphate (cGMP), mainly stimulated by nitric oxide in the penis corpus cavernosum. PDE5Is cause vasodilation in the penis by blocking the metabolism of cGMP, resulting in prolongation of the action of mediators of vasodilation, including nitric oxide (NO; [79]). These molecules are typically ingested 30 min–4 h before possible sexual intercourse. Typically, no more than once daily use is recommended. Out of all these molecules, tadalafil is associated with the longest half-life (e.g., 17 h), hence likely to be the molecule most frequently misused and associated with misuse and, at times, priapism events [80]. Some 2919 Spanish MSM subjects were surveyed; the most frequently used drugs for sexualized drug use (SDU) were poppers (53.6%), cannabis (19.6%), and Viagra (12.2%) [72]. The use of PDE5Is associated with chemsex varies substantially across Europe and is particularly common in the Netherlands and the United Kingdom [21]. Overall, they may be perceived by users as a preventive medication since specific chemsex-related molecules may cause erectile difficulties.

Rationale for use: increased erectile rigidity, counteracting the effects of drugs/alcohol that may attenuate erection [21], and increased erectile sensation; furthermore, with PDE5Is, there may be indirect effects such as increased libido, enhanced self-esteem, decrease performance anxiety, and impress/satisfy sexual partner(s) [55,81].

Risks: Those PDE5Is that are purchased from the web may not contain the declared substance and/or in the reported amount [82]. This may be associated with health safety risks [55]. Furthermore, cardiovascular-related issues may emerge from the association between PDE5Is’ vasodilatation effects and the adrenergic/serotoninergic properties of stimulants concurrently ingested during chemsex [29].

### 3.8. Popular Recreational Drugs: Cannabis and Cocaine

Cannabis and cocaine use is associated with sexual at-risk behavior [83]. Some 69 volunteers from the LBGTQIA+ French community were surveyed using both hair analysis and a questionnaire administration. On the 219 hair segments analyzed, the most commonly used drug was cocaine (e.g., 68% of cases [84].

Conversely, Vallee [85] examined the UK Biobank cohort (e.g., 115,604 participants), both cannabis use and alcohol consumption were significantly associated with an increase in the number of different sexual partners. Higher levels of sexual activity in cannabis users were recently confirmed by [86], who suggested that lower doses of cannabis may be linked to heightened sexual desire and enjoyment, whereas higher doses may lead to a decrease in sexual desire and performance.

Rationale for use: Cocaine use is largely associated with at-risk sexual behavior, possibly through an increase in sexual desire [87]. In preclinical models, cocaine is initially associated with contractions in cavernosal tissue strips persisting for several hours. However, chronic use of cocaine may be associated with reducing levels of noradrenalin sympathetic nerve due to an increased exposure to induced catabolic enzymes at the presynaptic-cleft level terminals, leading to priapism [88].

Conversely, a sexualized cannabis intake may be associated with relaxation, sensitivity, intensity, desire, different time perception, and disinhibition [89].

Risks: In association with acute cocaine intake, a vast range of cardiovascular [90] and psychopathological [91] issues may be observed. Cannabis use may be associated, for some, with negative effects on sexual functioning, such as sedation, lack of focus, and an increase in distraction levels [89]. It is being debated if chronic marijuana use can also lead to erectile dysfunction [92]. Both cocaine and cannabis intake have been associated with suicidal behavior in specific at risk populations [40].

### 3.9. Alcohol

Voluntary acute intoxication, at low to moderate blood alcohol levels, induces anxiolytic and disinhibiting effects [93]. Hence, alcohol can be used to support sexual experimentation, facilitating the transgression of conservative or restrictive social and sexual norms [94]. At lower doses, alcohol enhances sexual arousal primarily by decreasing psychological inhibitions, whereas, in being a sedative, at higher doses, it suppresses physiological sexual responses [95]. Overall, alcohol’s impact on sexual behavior is influenced by a complex interplay of historical, mythological, cultural, religious, physiological, pharmacological, expectancy-related, personal, and legislative factors [95]. Although not typically classified as a typical chemsex substance, alcohol can nonetheless play a role in broader patterns of substance use and associated risky behavior [96].

Rationale for use: The pharmacokinetic of ethanol follows a curve whose effects can be anticipated; hence, one could argue that many individuals who consume alcohol may attempt to modulate these effects to achieve the desired outcomes during sexual encounters. Moreover, alcohol consumption is frequently combined with the use of other substances [97], resulting in additive or synergistic effects.

Risks: Both acute and chronic alcohol intoxication can affect multiple organ systems [98]. Alcohol exerts central depressant effects; an acute intoxication may be life-threatening [99]. Short- and long-term alcohol consumption is associated with a variety of sexual dysfunctions in both males and females [100], for example, lowering libido, reducing genital sensitivity, or delaying orgasms. Of particular concern is the toxicity of cocaethylene, a metabolite resulting from the concomitant use of ethanol and cocaine [101]. Alcohol may be associated with narrowing cognitive attention to immediate and salient environmental cues, thereby facilitating impulsive decision-making, disinhibition, and engagement in risky behavior [102]. Indeed, alcohol consumption may directly influence sexual decision-making and is a key factor in determining the intent to engage in unprotected sex [103]. In some cases, individuals may be coerced into drinking so that their capacity to negotiate sexual consent is impaired (Cornelius et al., 2024) [104]. In heterosexual populations, males usually consume more alcohol than females [105], with alcohol itself only rarely perceived as a sexualized substance by young male consumers [94]. Due to alcohol’s normalized status and a common tendency to focus more on the perceived risks of other substances, alcohol-related risks in sexual contexts may be underestimated or difficult to manage [94].

### 3.10. Other Agents Occasionally Used in Chemsex Practices

A range of further compounds have occasionally been reported as self-administered in association with chemsex practices, including:

(a) Benzodiazepines: apart from prescribing medicines, a vast range of potent, designer/“exotic”, molecules [106] have been described. One could argue that levels of sedation may help to overcome fear and reduce the impact of stress related to the sexual encounter(s). However, benzodiazepines can be associated as well with sexual dysfunctions [107], together with risks of oversedation, coma, and being involved in non-consensual sexual activities [108].
(b) Cantharidin (e.g., “Spanish fly” [109]): this is a popular compound derived from blister beetles; it is known for its alleged sexual stimulating properties. Some Spanish fly preparations may contain cantharidin in dosages, which can be associated with both gastrointestinal- and kidney-related disturbances [109].
(c) Tropicamide: misuse of this ophthalmic anticholinergic compound, producing short-acting mydriasis and cycloplegia, has been recently described in association with chemsex practices [110]. When misused, tropicamide is typically injected intravenously, often in combination with other psychoactive drugs. Tropicamide-related psychoactive effects include hallucinations and dysphoria associated with slurred speech, persistent mydriasis, hyperthermia, tremors, convulsions, psychomotor agitation, tachycardia, and suicidal ideation [111].
(d) Image- and Performance-enhancing drugs’ (IPEDs) concomitant intake is likely and may be underestimated; for example, the use of steroids has been reported among individuals participating in chemsex sessions [112].
(e) Psychedelics: Additionally, anecdotal reports highlight the use of psychedelics during sex, possibly in combination with other substances; [113] recently provided further insights into this topic.

### 3.11. Chemsex and Polydrug Exposure

Polydrug use is typically reported during 95% of chemsex sessions, with an average of 3.5 agents per session [71]. In the UK scenario, together with GHB/GBL, the following substances were implicated in fatality cases: cocaine (38%), alcohol (33%), amphetamines (29%), ecstasy (29%), diazepam (24%), ketamine (24%), and mephedrone (24%) [53]. Polydrug use may be associated with pharmacokinetic-related issues, major interactions may involve those molecules metabolized by CYP3A4 and CYP2C9. This may lead to a significant level of interactions, particularly with HIV medication [29].

### 3.12. Which Drugs Are Being Possibly Misused in Post-Chemsex Scenarios?

In the hours and days after having been involved in both drug-related [47] and chemsex parties [64], individuals may experience a range of psychopathological disturbances, including emotional instability, risk of psychosis, altered body perception, and risk of suicide (e.g., “lethal lust”, [114]. From this point of view, one could argue that chemsex enthusiasts may be at high risk of misusing a range of medications to counteract these unpleasant/untoward/negative subjective effects. These molecules may arguably include:

(a) Tapentadol: Recent anecdotal reports from South East England may suggest an overall increase in tapentadol, including at post-chemsex ingestion; this is being carried out to achieve levels of both sedation and relaxation. Tapentadol may be misused either on its own [115], or in combination. This may occur with only carisoprodol/Soma (“red apple”) or with alprazolam, as well (“holy trinity”; [116].
(b) Pregabalin: the molecule is approved in Europe for the treatment of epilepsy/partial seizures, neuropathic pain, and generalized anxiety disorder. When misused, pregabalin is considered an “ideal psychotropic drug” to achieve specific mindsets, including sedative effects, which are arguably useful at post-chemsex, mixed with euphoria, dissociation, and opiate-like relaxation [117]. Misuse of pregabalin, at dosages up to 3–20 times higher than the maximal dosage indicated [32], mostly seems to occur orally, but intravenous use, rectal “plugging”, and smoking have been reported as well. In parallel with increasing prescribing levels, a growing black market is currently being observed [118].
(c) Quetiapine: Reasons for abuse of atypical antipsychotics may include the desire to “feel mellow” [119]. However, only 100 mg IR formulations are typically considered for misuse after having been crushed and snorted. Quetiapine (“Q ball”) is similarly anecdotally considered to “come off the psychedelic trip” [120], with typical misusers being clients with a previous substance abuse history.
(d) Olanzapine: The molecule is being advised online as the “ideal trip terminator” after a drug binge. It is self-prescribed for a few days only at very high daily dosages, e.g., up to 50 mg [121].

## 4. Discussion

An updated narrative overview of the pharmacological, clinical pharmacological, and toxicity issues related to the variety of psychoactive substances in use in chemsex sessions and sexualized drug behaviour has been provided here. Most psychoactive drugs mentioned are characterized by a stimulant (e.g., amphetamine-type substances, synthetic cathinones, and cocaine) and/or a psychedelic/dissociative (ketamine, MDMA) activity. However, GHB/GBL are very popular molecules as well, which are considered in the chemsex scenario.

### 4.1. Associated Risks

The use of stimulants is likely related to the need to increase as much as possible both sexual arousal and performance [15]. Furthermore, the empathogenic/entactogenic activities of some ATSs’ “love drugs” facilitate the occurrence of “feeling closer/more intimate’ sensations. All these drug-elicited properties may be perceived by chemsex enthusiasts as being useful since these sessions may be long/very long-lasting and involve intimate encounters with a range of strangers, e.g., people that have never met before the session itself. Furthermore, GHB/GBL may well provide the user with a subjective sensation of disinhibition, hence facilitating meeting with a higher number of sexual partners and involvement in both group sex and condomless anal intercourse with random partners [52]. The frequent mention of ketamine as a chemsex drug could be explained by its potent analgesic/anesthetic properties, facilitating the potentially painful receptive anal intercourse and/or fisting experiences [77].

Overall, however, sexual activity over protracted lengths of time under the influence of potent stimulant/dissociative/disinhibiting drugs can result in rectal trauma or penile abrasions and a significant increase in the risk of transmission of sexually transmitted diseases [55].

Another issue of real concern that emerged here related to the possible occurrence of intense, acute, and chronic medical and psychopathological consequences associated with the ingestion of these molecules, which are most typically consumed in combination (e.g., 3.5 drugs per session; [71]. Indeed, polydrug exposure can facilitate the occurrence of a serotonergic syndrome, with its relating life-threatening characteristics [122]; drug–drug pharmacokinetics’ interaction, which may be particularly relevant with HIV medication [29]; and sympathomimetic overstimulation, due to synergistic pharmacological interaction [46].

In association with the recent, possibly intense, chemsex-related polydrug exposure, a number of psychopathological disturbances, including mood disorders, suicidal ideation, and psychotic signs and symptoms, have been reported [64,114]. These disturbing subjective psychological experiences may lead to further levels of drug abuse, focusing on molecules with both strongly sedating (opiates/opioids; gabapentinoids) or antipsychotic activities.

### 4.2. Treatment Approaches

Given the serious health harms associated with the above stimulant/dissociative/disinhibiting polydrug ingestion, one would wonder about the best treatment and management approach to be considered. Unfortunately, however, for the treatment of withdrawal/cravings relating to most of the molecules discussed here, including stimulants and ketamine, no specific “ad hoc” medications have been approved [65]. In acute cases, intensive supportive care may be necessary; effective treatments may include adequate hydration, which helps manage dehydration, prevent rhabdomyolysis, and reduce the risk of renal failure; benzodiazepines, for example, diazepam or lorazepam, if properly dosed, due to their favorable pharmacokinetics, which can also be useful against hyperpyrexia and seizures; antipsychotics, for acute psychotic episodes not adequately managed with benzodiazepines [123] or other sedative agents. Conversely, for the inpatient treatment of GHB/GBL detoxification, high dosages of both benzodiazepines and baclofen may need to be administered [124]. Of note, routine screening tests often fail to detect most of these substances, making it essential to use validated analytical methods for their detection [32].

As with other drugs, the treatment of harmful drug use may need to include a psychosocial approach. For stimulant users, a 45–60 min clinical intervention/structured motivational discussion may need to include an exploration of the individual’s characteristics of use, desired and unwanted effects of stimulant ingestion, and plans for behavioral changes (for a comprehensive overview of these issues, see Abdulrahim and Bowden-Jones, 2015) [65]. Since, in most cases, users would be polydrug users, these interventions will, however, not need to focus on a single molecule in isolation.

### 4.3. Prevention

Clinical pharmacists may have a role as well in contributing to better monitoring the levels of sexual behavior-related drug intake. From this point of view, results of a national additional risk minimization measures program, implemented to train pharmacists for a safe supply of non-prescription sildenafil in the UK, have recently been made available. Within this program, some 86% of patients were advised on how to take sildenafil correctly, and about 70% of patients confirmed that they had received advice on lifestyle modifications to manage their erectile dysfunction-related health risks [125].

From the diagnostic point of view, there are gaps and potential risks of confusion among clinicians; the Diagnostic and Statistical Manual of Mental Disorders, latest edition (DSM-5) lacks, in fact, a specific category for sexual addiction, whereas the International Classification of Diseases (ICD-11) recognizes the possibility of a compulsive sexual behavior disorder [126]. Diagnostic difficulties can be further complicated by the potential of transitioning between disorders, such as substance use disorders, to behavioral addictions, with chemsex practices occurring intermittently or persistently during these transitions. Moreover, some authors suggest that certain personality traits and disorders, such as borderline personality disorder, may reflect a broader coping strategy where individuals prioritize reproductive traits and behavior as a way to deal with adversity [127].

Finally, the increasing application of computational modeling and AI-driven predictive tools has opened new avenues for identifying and monitoring emerging psychoactive substances, including those used in chemsex practices. As demonstrated in recent studies, structure-based and ligand-based approaches have successfully anticipated the appearance of novel substances before their formal identification in forensic or clinical settings [128]. These methodologies can be extended to a wide range of drug classes, including synthetic stimulants, hallucinogens, and dissociative agents. With AI-driven molecular design and screening, researchers can now explore vast chemical spaces with unprecedented speed and accuracy, potentially forecasting the next generation of designer drugs. This technological advancement underscores the need for regulatory agencies, toxicologists, and healthcare professionals to integrate AI-assisted tools into early warning systems.

### 4.4. Limitations

In order to fulfill the purpose of this review, presenting with a focus on a very specific topic, with most of the studies being relatively novel, a narrative approach has been preferred. This choice, which made making explicit the search strategy, has anyway provided broader literature coverage and more flexibility. We acknowledge, however, that this design has introduced a number of limitations. First, some of the studies included here did not disclose explicit criteria for article selection, which could lead to potential selection bias. Second, the review strategy employed led to the inclusion of studies with highly heterogeneous designs, methodological quality, and standardization of the methods used to characterize the impact of specific substances, including alcohol, on sexual behavior. Some of the included studies were small-sized, and, therefore, it remains unclear whether the findings could be generalized to the entire chemsex enthusiasts’ population.

## 5. Conclusions and Future Directions

Addressing the health harms associated with chemsex remains challenging due to the lack of updated and contextualized epidemiological data. Effective health policy interventions must be informed by comprehensive data on the patterns and consequences of chemsex-related drug use. Additionally, targeted media and social campaigns are essential to reduce the stigma that prevents individuals, particularly from the LGBTQIA+ community, from accessing necessary healthcare and support services [129,130]. Improving access to harm reduction programs, increasing awareness of chemsex-related risks, and integrating pharmacological and psychosocial treatment approaches are critical steps. Enhanced training for pharmacists and healthcare professionals, along with the use of AI-driven predictive tools for identifying emerging substances, can strengthen monitoring and early intervention efforts. A multidisciplinary approach that combines medical, psychological, and social support remains key to managing the complex challenges posed by chemsex-related drug use.

## Data Availability

No new data were created or analyzed in this study. Data sharing is not applicable to this article.
